# Evaluation of Cartilage Repair After Implantation of Labeled Human Chondrocytes as Free Cells or Spheroids in Rabbit Knees

**DOI:** 10.3390/ijms27146381

**Published:** 2026-07-17

**Authors:** Jacques Hernigou, Pascale Vertongen, Esfandiar Chahidi, Nathalie Gaspard, Jessica Lechanteur, Gaelle Lapouge, Joanne Rasschaert

**Affiliations:** 1 Laboratory of Bone and Metabolic Biochemistry, Université Libre de Bruxelles, 1070 Brussels, Belgium; pascale.vertongen@ulb.be (P.V.); joanne.rasschaert@ulb.be (J.R.); 2 Orthopedics and Traumatology Department, EpiCURA Hospital, 7331 Baudour, Belgium; 3 Orthopedics and Traumatology Department, CHU (Centres Hospitaliers Universitaires) Helora—La Louvière Hospital, 7100 La Louvière, Belgium; 4 Département D’Enseignement de la Biologie, Université Libre de Bruxelles, 1070 Brussels, Belgium; nathalie.gaspard@ulb.be; 5 Erasme Animal Facility, Université Libre de Bruxelles, 1070 Brussels, Belgium; gaelle.lapouge@ulb.be

**Keywords:** chondrocytes, spheroids, gold nanoparticles, rabbits, xenogenic

## Abstract

Articular cartilage repair remains a clinical challenge due to the tissue’s limited regenerative capacity. This study aimed to perform in vivo tracking of human chondrocytes after implantation in femoral trochlear defects. Moreover, we tested the rabbit xenogeneic model to assess the efficacy of human chondrocyte implantation, either in the form of cell suspension or in the form of spheroids, for cartilage regeneration. Chondrocytes were isolated from osteoarthritic human knees, expanded in vitro, and labeled with gold nanoparticles (AuNPs) to enable their in vivo tracking. AuNP labeling did not impair chondrocyte viability and spheroid formation. Knee osteochondral defects were then treated with the labeled cells, either injected intra-articularly as single cells in suspension or aggregated into scaffold-free spheroids and further implanted. Two weeks post-transplantation, macroscopic and histological evaluations were performed to assess cartilage repair and cell engraftment. The presence of labeled cells was detected only in spheroid-treated lesions that exhibited significantly greater tissue filling as reflected by O’Driscoll and ICRS scores. Moreover, localized expression of type II collagen was observed in some of the spheroid-treated defects. In contrast, single cell intra-articular injection resulted in poor cartilage repair and the absence of labeled cells at the defect site. Altogether, our results indicate that human chondrocyte delivery in the form of scaffold-free spheroids in rabbit knees enhances their retention at the defect site and supports early stages of cartilage regeneration. Moreover, our results suggest that the xenogeneic rabbit model could be a useful platform for evaluating human-derived cell therapies.

## 1. Introduction

Articular cartilage is a specialized tissue capable of withstanding the repetitive forces that the knee joint experiences during everyday activities, such as walking and climbing stairs [1]. Due to its lack of blood supply and unique structure, the articular cartilage is vulnerable to irreversible injury, often leading to degenerative conditions like osteoarthritis (OA). Indeed, due to its avascular nature, oxygen and nutrient supply to the articular cartilage relies on diffusion from surrounding tissues, resulting in low cell proliferation and reduced regenerative capacity [1]. Various therapeutic strategies have been explored to address these biological deficiencies, with the most recent efforts focusing on regenerating cartilage using cells from the musculoskeletal system [2].

Bone marrow stimulation, cell-based techniques using mesenchymal stem cell or chondrocyte implantation, and osteochondral grafting have shown good clinical results in treating cartilage defects [3,4,5]. However, all of these approaches, with the exception of the last one, predominantly lead to the formation of fibrocartilage rather than hyaline cartilage, which compromises the long-term durability and the biomechanical properties of the repaired tissue [3]. Osteochondral grafting remains up to now the only technique capable of restoring native hyaline cartilage architecture, but its therapeutic application is limited by the morbidity associated with donor site harvesting, the availability of suitable grafts, and the size of the defect to treat [3,6].

The use of chondroprogenitors (CPs), the resident cells of cartilage with stem cell-like characteristics, has emerged as a promising alternative due to the intrinsic chondrogenic commitment of the CPs, although their clinical applicability is still under validation [7].

All of the techniques described above, as well as new therapies for articular cartilage repair, fall into four categories: scaffold-only approaches, cell-based methods in the absence or presence of an exogenous scaffold, and more recently, scaffold-free spheroids [2,6,8].

Scaffold-free spheroids, usually called spheroids, refer to “autologous matrix-associated chondrocytes” [9,10]. They are spherical clusters of ex vivo expanded human chondrocytes and their self-produced (autologous) extracellular matrix. As another advantage, spheroids naturally adhere to the subchondral bone, thus eliminating the need for sutures, membranes, covers, or glue after their implantation [9,10,11].

Despite the increasing clinical use of chondrocyte- and CP-based therapies, the fate of the implanted cells, their longevity within the repair tissue, and their optimal mode of delivery remain poorly defined. As a result, in vivo cell tracking approaches are needed to determine whether transplanted cells persist within the repair site and how different administration techniques, including intra-articular injection of individual cells, influence their in vivo distribution and survival as well as cartilage repair.

Testing human chondrocytes in a controlled experimental setting requires a xenogeneic model, as no experimental equivalent exists for evaluating human cells in vivo otherwise. We therefore transplanted AuNP-labeled human chondrocytes into osteochondral defects of rabbit femoral trochleae, either as free cells or as scaffold-free spheroids. This approach is further supported by the unique immunological status of articular cartilage: as an avascular and alymphatic tissue, cartilage benefits from a degree of immune privilege [12], and the immunological response to transplanted chondrocytes—particularly in a xenogeneic context—remains poorly characterized.

The present study thus assessed the in vivo efficacy of osteochondral tissue regeneration after xenogeneic chondrocyte implantation, either in the joint or in the subchondral bone. Human chondrocytes, derived from surgical remnants of total knee arthroplasty (TKA), were thus AuNP-labeled and transplanted into osteochondral defects of rabbits’ knees, either as free cells or in the form of spheroids. We used the rabbit model as compared to rodents, rabbits share a closer phylogenetic and anatomical relationship with humans [13,14], and the defect size that can be created in their knees facilitates spheroid transplantation without causing defect overcrowding. Furthermore, we thought that short-term studies could be conducted on rabbits without needing immunosuppressive treatment [15].

## 2. Results

### 2.1. Characterization of Chondrocytes Obtained from TKA

On average, 2.03 ± 0.17 (*n* = 15) million viable cells were collected per gram of tissue, allowing for the consistent expansion of chondrocytes and their subsequent implantation.

Cells obtained after cartilage digestion were plated (P0) and analyzed up to P3 in terms of doubling time (DT), mRNA expression of ECM, and up to P2 for phenotypic markers to confirm the chondrogenic nature of the isolated cells.

The population doubling time (DT) of chondrocytes cultured in medium supplemented with 10% human serum was stable throughout in vitro expansion from P0 to P3, with a mean value of 4.4 ± 0.21 days (*n* = 11; no statistical difference between passages—*p* > 0.05). This observation indicates that the isolated cells retained their proliferative potential during early passages.

**Figure 1:**
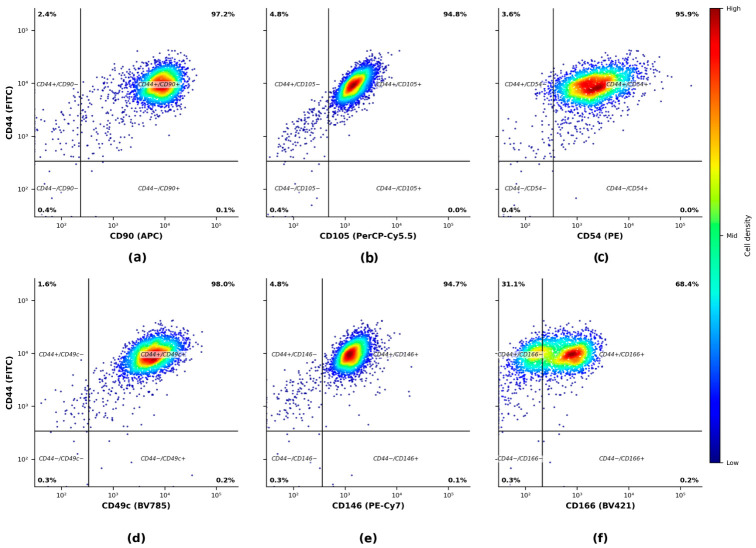
(**a**–**f**) Representative flow cytometry plots of P0 chondrocytes cultured in 10% human serum (*n* = 2). The plots depict the co-expression of CD44 with specific surface markers, identifying subpopulations as follows: CD44^+^/CD90^+^ (**a**), CD44^+^/CD105^+^ (**b**), CD44^+^/CD54^+^ (**c**), CD44^+^/CD49c^+^ (**d**), CD44^+^/CD146^+^ (**e**), and CD44^+^/CD166^+^ (**f**).

Flow cytometry was used to assess the expression of surface markers up to P2 to monitor phenotypic stability and detect signs of dedifferentiation during monolayer culture (Figure 1).

Chondrocytes consistently exhibited high expression of the mesenchymal markers CD90 and CD105 (>90%), as well as of the chondrocyte-associated markers CD44 and CD54, across all passages (Figure 1a–c). CD49c and CD146, both involved in cell adhesion and cell–matrix interactions, remained strongly expressed (>90%) across all passages; while CD49c (integrin α3) primarily mediates interactions with the ECM, CD146 (MCAM) is also associated with mesenchymal progenitor-like features and may contribute to the maintenance of a chondrogenic potential (Figure 1d,e).

In contrast, CD166 (ALCAM), a marker associated with mesenchymal stem cells and chondroprogenitors, showed a distinct expression pattern, being detected in approximately 60% of the cell population from P0 to P2 (Figure 1f).

We did not observe the expression of CD34 or CD45 at any cell passage, confirming the absence of hematopoietic cell contamination.

Of note, although the experiments were conducted on cells of two independent donors cultured in the presence of 10% human serum, we observed similar marker expression patterns with cells cultured in the presence of 5% human serum from P0 to P2 (*n* = 7).

**Figure 2:**
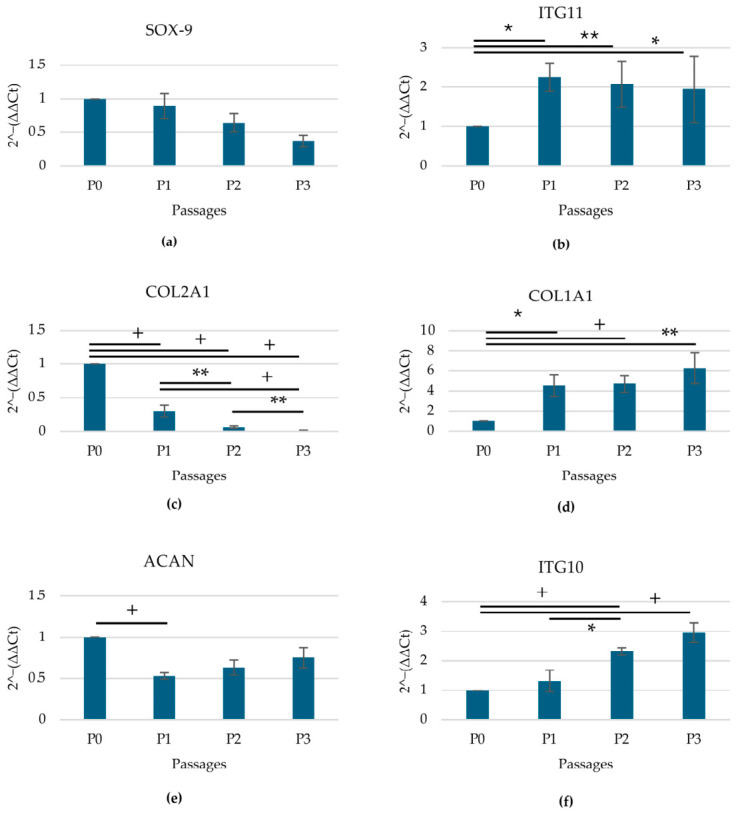
(**a**–**f**) Quantitative gene expression analysis of ECM components in chondrocytes at passages P0 to P3 (*n* = 9). * *p* < 0.05; ** *p* < 0.01; ^+^ *p*< 0.005.

The transcriptional profile of key ECM components was analyzed from P0 to P2 to characterize the chondrocytes and evaluate their genotypic stability during expansion. qPCR was performed to assess the expression of SOX-9, COL2A1, ACAN, ITG11, COL1A1, and ITG10 (Figure 2). When examining gene expression changes across passages, SOX-9 expression seemed to decrease from P0 to P3, although this variation did not reach statistical significance (Figure 2a). In contrast, COL2A1 expression significantly declined with increasing passages (*p* < 0.05; Figure 2b), indicating a marked loss of cell ability to express hyaline cartilage, a characteristic of mature chondrocytes. ACAN expression significantly decreased between P0 and P1 (*p* < 0.005) and then remained stable (Figure 2c). Conversely, ITG11 expression significantly increased at P1 (*p* < 0.05) (Figure 2d), confirming the shift toward a fibroblastic state. Similarly to ITG11, COL1A1 expression was also found to increase (*p* < 0.05—P1 vs. P0; Figure 2e). In contrast, ITG10 expression increased at P2 and P3 (Figure 2f).

**Figure 3:**
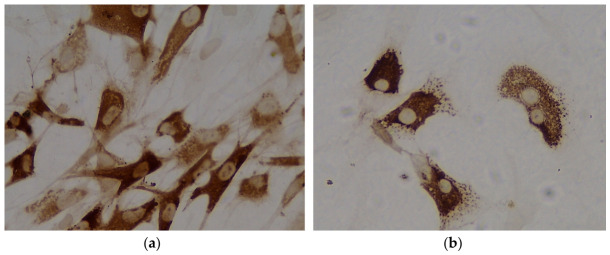
(**a**,**b**) Representative immunohistochemical staining of P1-expanded chondrocytes (*n* = 3) showing positive labeling (brown staining) for ACAN (**a**) and COL2A1 (**b**). Zoom = 40×.

We further examined the expression of ACAN and COL2A1 at the protein level by IHC analysis. Positive staining for both chondrocyte markers was observed at P1, indicating that those chondrocytes retained the ability to produce key ECM components characteristic of the hyaline cartilage phenotype (Figure 3a,b).

Overall, the characterization of chondrocytes derived from enzymatically digested human cartilage revealed that the isolated cells exhibited a stable proliferative capacity up to P3. Moreover, cells retained several defining features of the chondrocytic lineage during the first passage in monolayer culture. A transcriptional profile indicative of dedifferentiation—marked by the progressive loss of COL2A1 and increased expression of COL1A1—was then observed.

### 2.2. Efficiency of AuNP Transfection in Chondrocytes

**Figure 4:**
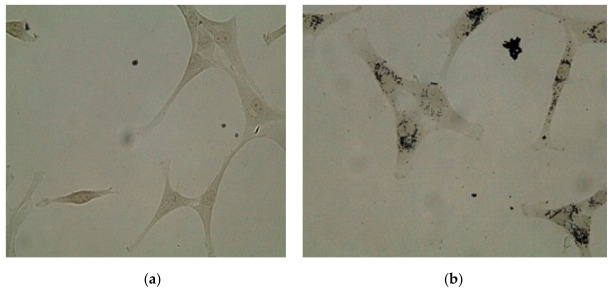
(**a**) P1 chondrocytes without AuNP transfection. (**b**) P1 transfected chondrocytes showing the presence of intra cellular AuNPs that were mainly localized around the nucleus (zoom = 40×).

The AuNP transfected chondrocytes were examined using light microscopy after the silver enhancement method, providing reliable visualization of AuNP internalization and allowing for a semi-quantitative assessment of transfection success. Microscopic analysis revealed that AuNPs were predominantly localized near the perinuclear region, as described in the literature [16] (Figure 4). Chondrocyte examination under bright-field microscopy revealed that approximately 60% of the chondrocytes had internalized AuNPs, while 20% were negative, and 20% displayed ambiguous or diffuse labeling (*n* = 3).

Cell viability was assessed one to 96 h after transfection in labeled versus unlabeled chondrocytes and was not affected by the presence of AuNPs at each time point (*p* > 0.05; *n* = 3) (Appendix A—Figure A1). Surface marker expression was assessed by flow cytometry (FACS) to compare the expression profile of labeled and unlabeled chondrocytes. We did not observe differences between the two cell populations (Appendix B—Figure A2). This analysis was performed only once, as no discrepancies were detected between groups and as the cell quantity required for FACS analysis precluded further use of the donor’s cells for subsequent spheroid formation and implantation.

### 2.3. Evaluation of Spheroids

**Figure 5:**
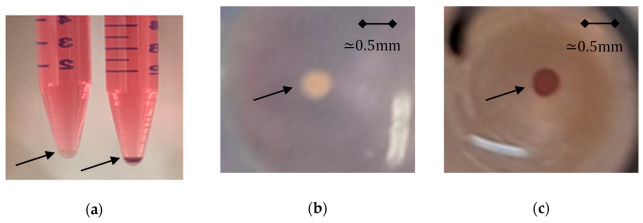
(**a**) Cell pellet (arrows) of unlabeled (white) and AuNP-transfected chondrocytes (black). (**b**) Picture of a spheroid formed from unlabeled chondrocytes: white macroscopical aspect. (**c**) Picture of a spheroid formed from AuNP-labeled chondrocytes: black macroscopical aspect.

Spheroids were generated from 150 to 200 × 10^3^ P1 human articular chondrocytes collected and seeded on coated non-adherent 96-well plates. Given the substantial cell number required for in vivo experimentation and the onset of dedifferentiation beyond the first passage—evidenced by the marked reduction in COL2A1 (Figure 2b) and increase in COL1A1 (Figure 2e) gene expression—the use of P1 chondrocytes represented an optimal compromise for AuNP transfection followed by spheroid formation. Notably, spheroids formed from AuNP-transfected chondrocytes did not exhibit major visual alterations in morphology when compared to those derived from non-transfected chondrocytes. The only detectable difference was a darkened pigmentation of the labeled cells and spheroids, resulting from the internalization of AuNPs (Figure 5a–c).

**Figure 6:**
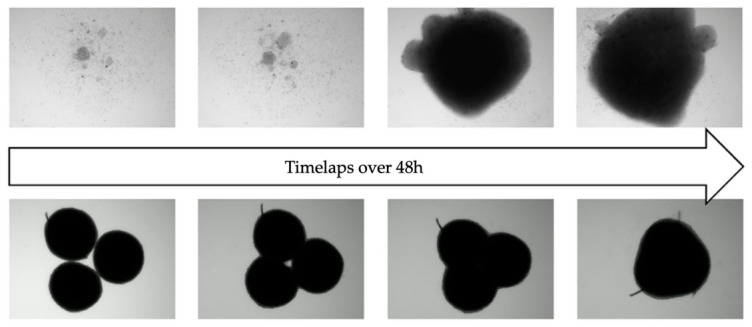
Top timeline: Formation of an individual spheroid from 200 × 10^3^ plated chondrocytes over 48 h. Bottom timeline: Fusion of 3 individual spheroids in one macro spheroid of around 600 × 10^3^ chondrocytes.

The intracellular presence of AuNPs did not visually affect spheroid formation as the AuNP-labeled chondrocytes were still able to fuse (Figure 6; Supplementary Materials—Video S1; Video of the formation of a spheroid over 48 h). Importantly, spheroids maintained their pigmentation over several days of culture, suggesting no or minimal loss of AuNP labeling. Moreover, the kinetics and rate of spheroid formation, as well as the resistance of the spheroids to mechanical manipulation, seemed comparable between the non-transfected and transfected chondrocytes.

Following the initial cell aggregation period in non-adherent culture conditions, three spheroids formed from AuNP-labeled cells were successfully merged to generate a larger spheroid comprising approximately 600 × 10^3^ cells (Figure 6; Supplementary Materials—Video S2; Video of the fusion of 3 spheroids over 48 h).

**Figure 7:**
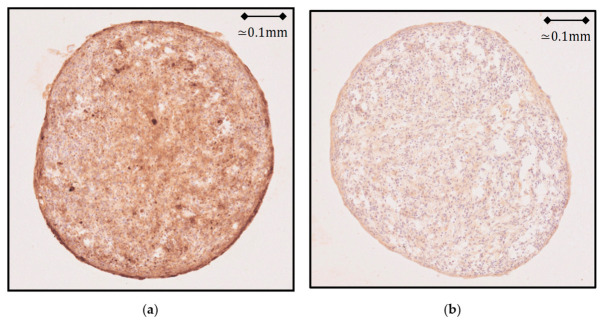
(**a**) Immunohistochemical positive staining for ACAN in an unlabeled spheroid (homogeneous brown staining). (**b**) Immunohistochemical negative staining for COL2A1 in an unlabeled spheroid, with signal intensity indistinguishable from background noise (*n* = 3).

To assess the capacity for extracellular matrix production, spheroids were fixed, paraffin-embedded, sectioned, and subjected to immunohistochemical staining for aggrecan and COL2A1, two key components of cartilage-specific ECM. Positive aggrecan staining was observed in unlabeled spheroids (Figure 7a).

Regarding COL2A1 expression, our results demonstrated no immunohistochemical labeling for this cartilage-specific ECM (Figure 7b).

### 2.4. Rabbits’ Post-Operative Evolution After Xenogeneic Transplantation of Human Chondrocytes

All animals recovered from surgery and were monitored daily for general condition, weight, wound healing, and pain scores according to NC3Rs guidelines. Two rabbits (one in the experimental and one in the control groups) developed minor wound dehiscence at the surgical site within the first postoperative week. These cases were managed by brief re-anesthetization and surgical debridement.

In terms of locomotor recovery, moderate lameness was observed in all animals, with an average resolution around day 10 (D10). No animals displayed persistent or severe gait disturbances thereafter.

Across the cohort, all rabbits experienced a transient postoperative weight loss, which was most pronounced between postoperative day 1 (D1) and days 4–5 (D4–D5). After this period, weight stabilized with mild oscillations, and at the time of sacrifice (D15), most animals had regained their baseline weight, with an average deficit of less than 50 g compared to their preoperative value (Appendix C—Figure A3).

The NC3Rs clinical score, used to assess signs of pain or distress, was moderately elevated during the first 48 h following surgery, primarily due to reduced mobility and grooming activity. These signs resolved spontaneously, and the score normalized thereafter. Importantly, no differences in clinical evolution were observed between animals treated with spheroids and those receiving intra-articular chondrocyte injections, suggesting that both procedures were equally well-tolerated from a postoperative standpoint.

### 2.5. Repartition of Implanted Cells

In the control rabbit group receiving an intra-articular injection of AuNP-labeled free chondrocytes, no AuNP-positive cells were microscopically identified in the defect area (Appendix D—Figure A4). This observation suggested the absence of chondrocyte engraftment at the defect site following their intra-articular delivery and was concomitant to the absence of detectable cartilage regeneration in these animals.

**Figure 8:**
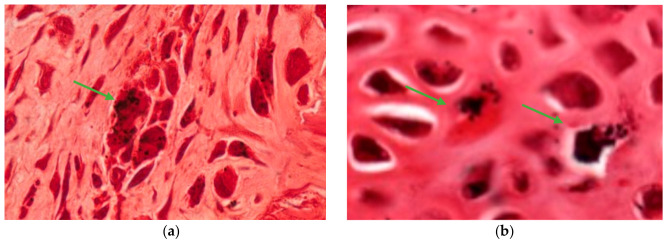
(**a**,**b**): Presence of AuNP-positive cells (black particles—green arrows) in the repair tissue of a spheroid-treated defect (Safranin-O staining). (**a**) Zoom = 40×; (**b**) zoom = 80×—immersion.

In the rabbit experimental group, the presence of labeled cells was microscopically observed within the repair tissue in 6 (out of 7) treated defects, indicating successful spheroid retention and engraftment at the implantation site (Figure 8). Notably, the only spheroid-treated defect that did not show AuNP labeling—indicating the absence of retained human chondrocytes at the repair site—displayed a poor O’Driscoll score (7 points /23), as well as its adjacent untreated defect (7 points /23).

Quantitative cell-counting analysis, performed on all available histological sections, corroborated the qualitative observations described above. In the spheroid-treated defects, approximately 5% of cells were AuNP-positive, whereas no labeled cells were observed in the adjacent untreated lesions (internal control) of the experimental rabbit group, supporting the localized engraftment of spheroid-based implantation.

In summary, these findings underscore that chondrocyte implantation in the form of spheroids is more efficient than their intra-articular injection as free cells for their retention at cartilage defect sites, leading to enhanced cellular engraftment.

### 2.6. Evaluation of Defect Filling and Cartilage Repair

**Figure 9:**
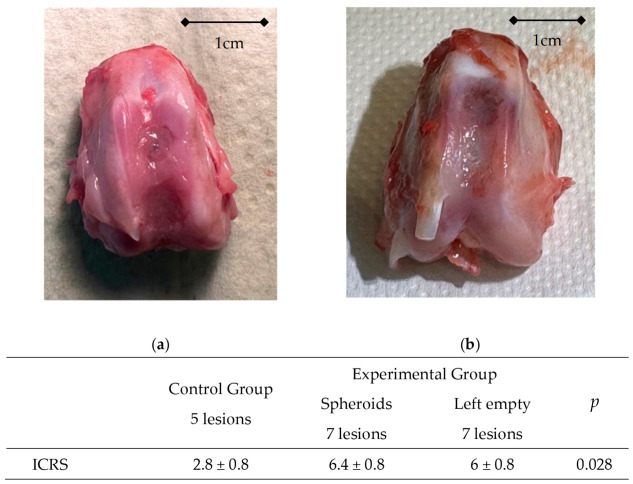
Representative macroscopic evaluation of two knees 15 days after surgery. (**a**) Poor defect filling representative of the control group. (**b**) Defect repair with newly formed tissue, representative of the experimental group.

**Figure 10:**
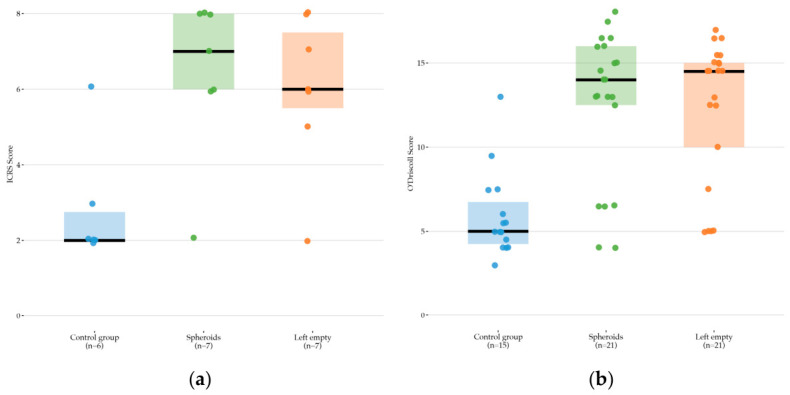
Individual ICRS and O’Driscoll histological scores by treatment group. (**a**) ICRS scores—each dot represents one defect. (**b**) O’Driscoll scores—each dot represents one histological section. The horizontal black bar indicates the median, and the colored area represents the interquartile range.

In the rabbit control group, we excluded one defect from the analysis because it was not drilled to the intended depth intraoperatively; histological examination indeed showed normal hyaline cartilage with no evidence of defect creation. Therefore, five control defects were used for the histological analysis. The mean macroscopic ICRS score was 2.8 ± 0.8, indicative of poor defect filling and compromised surface integrity (Figure 9a and Figure 10a).

In contrast, in the experimental rabbit group treated with AuNP-labeled spheroids, the mean ICRS scores reached 6.4 ± 0.8 for spheroid-treated defects and 6.0 ± 0.8 for untreated defects, reflecting moderate surface repair. No significant difference was observed between the two sites within the same joint (Figure 9b and Figure 10a). Notably, the outcomes observed in the control group were significantly inferior to those of the experimental rabbit group, both for spheroid-treated defects (*p* = 0.0002) and for untreated defects (*p* = 0.0003).

**Figure 11:**
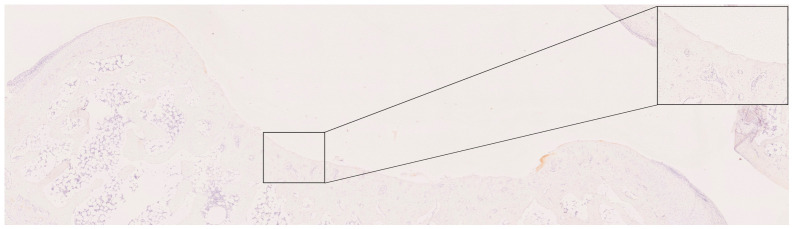
Representation of no filling—no repair in cartilage defect (intra-articular injection of AuNP-chondrocytes). Zoom 20×.

Histological analysis of the rabbit control group defects revealed a mean O’Driscoll score of 5.9 ± 0.7, consistent with disorganized tissue architecture, low cellularity, and the absence of cartilage-specific ECM features (Table 1 and Figure 10b). In that group, 80% of the defects showed no filling, indicating a lack of cartilage repair in all three animals (Table 1; Figure 11). Notably, the mean O’Driscoll score was significantly lower than those observed in the experimental group for both spheroid-treated and untreated defects (*p* = 0.00011).

**Figure 12:**
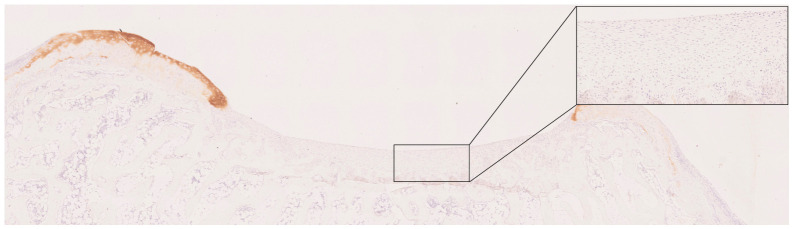
Representation of fibrocartilage repair in cartilage defect. Zoom 20×.

**Figure 13:**
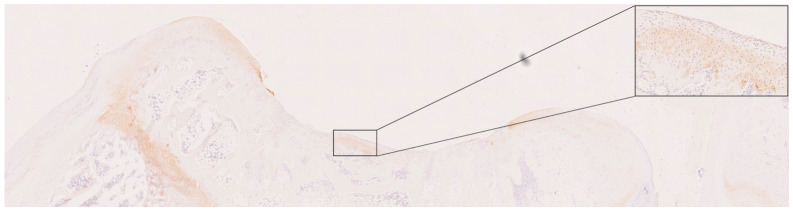
Representation of partially differentiated repair in cartilage defect (defect treated with spheroids). Zoom 40×. Brown staining indicates type II collagen (COL2A1) immunohistochemistry.

In the experimental group, 71% of the rabbits exhibited evidence of repair in both the spheroid-treated and untreated adjacent defects. Spheroid-treated defects demonstrated fibrocartilage formation in 42.8% of cases and hyaline-like cartilage in 28.6% (two rabbits), characterized by chondrocyte-like cells embedded in a COL2A1-positive matrix (Table 1; Figure 12 and Figure 13). The mean O’Driscoll score for these defects was 12.6 ± 0.96.

Similarly, defects left empty within the same group exhibited fibrocartilage in 71% of cases, with a mean O’Driscoll score of 12.4 ± 0.9 (Table 1; Figure 12). No significant differences were observed between spheroid-treated and untreated defects in terms of histological classification or O’Driscoll scores.

Overall, the absence of significant differences between the treated and untreated defects within the same joint suggests a local paracrine effect, whereby bioactive factors or extracellular vesicles released from the spheroid-treated site may influence the repair response in adjacent lesions.

## 3. Discussion

To our knowledge, this study is the first to demonstrate that human spheroids labeled with AuNPs can engraft and favor early fibrocartilage or mixed “fibro and hyaline” cartilage regeneration in a rabbit xenogeneic model in the absence of immunosuppressive treatment.

Our results collectively demonstrate that xenogeneic chondrocyte implantation in the form of spheroids was more effective than an intra-articular injection of chondrocytes in suspension to treat knee cartilage defects. In the spheroid-treated defects, we observed AuNP-labeling that was concomitant to filling of the defect, higher macroscopic repair scores, and improved structural integration. Moreover, we showed the presence of COL2A1 in localized regions of the articular defect in two rabbits of the experimental group treated with spheroids, indicative of an early-stage hyaline-like cartilage regeneration. In contrast, in the group receiving free chondrocytes, we did not detect AuNPs at the defect site after sacrifice, and both macroscopic and histological evaluations revealed unfavorable outcomes, with no evidence of tissue repair.

The choice of the rabbit as an experimental model rested on several anatomical, genomic, and immunological characteristics that collectively make it particularly suited to the evaluation of human-derived cartilage repair strategies. From an anatomical point of view, its joint size offers a favorable balance between being large enough to allow for precise surgical manipulation and defect creation while remaining manageable for housing and handling compared to larger animal models. Moreover, the rabbit knee exhibits structural similarities to the human knee and comparable cartilage thickness relative to joint size, enabling relevant biomechanical loading patterns [17]. Importantly, the rabbit is one of the most extensively studied species in the field of cartilage research, with a rich body of literature describing the natural history and progression of cartilage lesions, spontaneous healing patterns, and responses to experimental repair strategies [18,19].

Additionally, the rabbit genome shows high homology with the human genome in genes relevant to cartilage biology and repair, which is particularly advantageous in a xenogeneic transplantation setting, as it may facilitate better cellular integration and reduce interspecies incompatibility in signaling pathways [13,14,17]. From an immunological perspective, no adverse reactions or signs of rejection were observed. While articular cartilage is often considered immune-privileged, long-term xenogeneic studies have reported variable outcomes, ranging from tolerance to delayed immune responses [20,21,22].

The 15-day endpoint was selected to address three complementary considerations. First, from an immunological standpoint, this timeframe captures early host responses to xenogeneic implantation—a critical window for assessing short-term tolerance without immunosuppression. Second, it ensures the reliable detection of AuNP-labeled cells before signal degradation. On the one hand, the stability of AuNP signals in spheroids may reflect the limited proliferative activity of chondrocytes in this configuration, as previously reported [23]. On the other hand, this apparent stability must be interpreted with caution, since Balfourier et al. (2020) demonstrated that intracellular AuNPs undergo progressive lysosomal breakdown beginning within a few weeks, which could compromise the accuracy of long-term cell tracking [24]. Consistent with this short timeframe, our histological findings correspond to an early stage of tissue repair. Although complete hyaline cartilage restoration was not expected within this period, the focal expression of COL2A1 observed in 28% of the spheroid-treated defects (2 out of 7) is consistent with the early-stage production of cartilage-specific matrix components [18]. Whether this focal COL2A1 expression represents an initiating step toward more mature cartilage tissue remains to be determined in longer-term studies.

Taken together, these findings highlight both the relevance of the rabbit xenogeneic model for early-phase evaluation of cartilage repair strategies and the importance of temporal considerations when interpreting regenerative outcomes and cell-tracking data. Long-term studies will be required to determine whether these early improvements translate into stable hyaline cartilage regeneration.

Previous studies have demonstrated that the expression of cartilage-specific markers, notably COL2A1, decreases markedly beyond P1 in monolayer culture, coinciding with the onset of chondrocyte dedifferentiation. This process is characterized by elevated COL1A1 expression and a shift toward a fibroblastic morphology [17]. Primary cultures (P0) generally yield an insufficient number of cells for large-scale experiments, thereby necessitating at least one passage of expansion. Importantly, P1 chondrocytes have been reported to retain chromosomal stability, with no detectable karyotypic alterations, ensuring genetic integrity for downstream applications [25]. In the present study, chondrocytes were expanded in the presence of human serum (HS) rather than fetal bovine serum (FBS), given that HS has been reported to better maintain the chondrocytic phenotype during in vitro expansion. Yin et al. (2025) indeed recently demonstrated that supplementation with 10% autologous HS more effectively preserved cell morphology, proteoglycan production, and COL2A1 expression compared to FBS [26]. Also, we generated spheroids from P1 chondrocytes seeded in non-adherent culture conditions, a strategic compromise for preserving the chondrocytic phenotype and obtaining a sufficient number of cells.

Our results of the mRNA expression of COL1A1 and COL2A1, SOX-9, as well as ACAN and ITG11, over successive passages in monolayer culture are consistent with the literature and supported our methodology [27]. At early passages (P0 and P1), the concomitant expression of COL2A1, SOX-9, and ACAN supports the chondrogenic identity of the cells, indicating that they correspond to articular chondrocytes or chondroprogenitor-like cells. With increasing passages, the observed upregulation of COL1A1 and ITG11, together with the progressive decrease in ACAN and COL2A1 expression, is indicative of chondrocyte dedifferentiation, consistent with previously described phenotypic drift in monolayer culture.

However, we observed that ITG10 mRNA expression increased at P2 and P3, an unexpected result that contrasts with the classical decrease of cartilage-specific markers during chondrocyte expansion [28]. A careful review of the literature reveals that the evolution of ITG10 expression in monolayer culture has not been cautiously investigated. Most available studies focus on ITG10 basal expression, developmental roles, or regulation during chondrogenesis, rather than passage-dependent dynamics [29,30,31]. Consequently, the discrepancy between our findings and existing paradigms could be due to a lack of reference data.

The combined expression of CD44, CD54, CD49c, CD90, CD146, and CD105, together with partial CD166 positivity, reflects a phenotype frequently reported in early-passage cartilage cells exhibiting culture-associated modulation toward a progenitor-like state. This observation is in agreement with previous studies showing that articular cartilage contains a subpopulation of progenitor cells (ACPCs) displaying mesenchymal stromal cell-like marker expression profiles, including CD105, CD166, and CD146 [32,33].

Importantly, such progenitor-like populations have been identified not only in healthy cartilage, but also in osteoarthritic (OA) cartilage, where they retain chondrogenic potential despite disease-associated alterations [32,33].

The biomechanical properties of chondrocytes are known to differ significantly between healthy and osteoarthritic cartilage, with OA cells displaying reduced stiffness and impaired viscoelastic responses as a consequence of ECM degradation and disrupted matrix homeostasis [34]. In the context of cartilage repair, the capacity of implanted cells to respond to mechanical stimuli and engage mechanotransductive pathways driving matrix synthesis represents an important determinant of long-term repair quality. The retention of a progenitor-like profile in our expanded OA-derived chondrocytes, as evidenced by sustained COL2A1 and ACAN expression, suggests that the cells retained sufficient chondrogenic competence to participate in mechanobiologically relevant repair processes following implantation.

These findings suggest that the phenotype observed in our study may reflect, at least in part, the presence of cartilage-derived progenitor-like cells during in vitro expansion, rather than a purely differentiated chondrocyte population. Critically, this progenitor-like profile did not come at the expense of chondrogenic identity: the concomitant expression of ACAN and COL2A1 at the mRNA level, confirmed by positive immunohistochemical staining, indicates that the cells retained their chondrogenic features throughout expansion—supporting their suitability for implantation in a cartilage repair setting.

Cell labeling with AuNPs constituted the final step of our cell preparation protocol, before spheroid production. We used 10 nm diameter AuNPs to label chondrocytes. The scientific literature indicates that AuNPs in the 10–30 nm diameter range display minimal cytotoxicity and negligible effects on proliferation and gene expression in various primary cell types, including chondrocytes and mesenchymal cells [35]. Based on the manufacturer’s specifications (7.1 × 10^12^ particles/mL), the amount of 10 nm AuNPs used for cell labeling in our study corresponds to an effective concentration of approximately 0.18 nM. This is several orders of magnitude lower than those reported to induce cytotoxic or pro-inflammatory effects in chondrocytes. Notably, Pascarelli et al. described deleterious effects and alterations in gene expression only at concentrations ≥ 160 µM—a threshold approximately 900,000-fold higher than the concentration applied in the present study [36].

Previous studies reported improved outcomes using spheroid-based strategies in articular cartilage repair [37,38,39,40]. Several factors may explain the superior performance of spheroids. It has been shown that their 3D architecture provides a supportive microenvironment for cell–cell interactions, maintains chondrocyte viability and genotype, and facilitates natural adherence to the defect site without requiring adhesives or surgical fixation [8,25,41,42]. In addition to favoring local retention, the structured delivery promotes better spatial organization of the implanted cells [43], two critical factors for sustained tissue regeneration. In our hands, we did not detect any in vitro synthesis of COL2A1 within the spheroids themselves. This observation is in line with some recent reports showing low or delayed COL2A1 expression in certain spheroid systems under specific culture conditions, particularly when expanded or partially dedifferentiated chondrocytes are used [44]. However, older studies from the 1980s–1990s, as well as a more recent work by Schubert and Anderer, have clearly demonstrated the presence of COL2A1 within spheroids under chondrogenic conditions, indicating that they might be capable of re-expressing a hyaline-like matrix profile in vitro under specific culture conditions [11,45].

Notably, the defects left untreated within the same joint as the spheroid-implanted experimental group also exhibited some degree of cartilage repair, albeit characterized exclusively by fibrocartilage tissue. In contrast, defects in the control group receiving an intra-articular injection of free chondrocytes showed no evidence of healing. Such a differential outcome could suggest that spheroids may contribute to cartilage repair not only through direct engraftment of implanted chondrocytes, but also by exerting local paracrine effects that might stimulate chondroinduction in adjacent untreated lesions. It is noteworthy that the one animal in which no AuNP labeling was detected at the spheroid-treated defect also displayed a poor O’Driscoll score at the adjacent untreated defect (7/23). While this single observation cannot support any mechanistic conclusion, it is consistent with the overall pattern observed across the group and warrants further investigation. Previous experimental evidence have indeed shown that spheroids may function as biological organizers rather than mere cellular fillers by releasing a broad spectrum of trophic signals—including cytokines, growth factors such as BMP-2, and extracellular vesicles (EVs)—that can diffuse into adjacent cartilage defects and modulate the surrounding regenerative microenvironment [46]. Altogether, these signals could further facilitate the recruitment of host cells, promote chondrogenic differentiation, and ECM remodeling, even at sites distant from the implanted spheroids [47,48,49,50]. This hypothesis aligns with the emerging model of indirect regeneration wherein transplanted cells exert their effects not solely through direct tissue integration but also by modulating the local regenerative niche. However, since no paracrine mediators were measured in the present study, this remains a hypothesis that would require dedicated experimental validation.

Taken together, these findings indicate that spheroid-derived chondrocytes may not be the sole contributors to tissue repair, but rather, could participate in a cooperative regenerative process involving both donor and host-derived cells, as suggested by the coexistence of labeled and unlabeled cells within the regenerated tissue. Indeed, the presence of both AuNP-labeled and unlabeled cells within the regenerated tissue is consistent with the possibility that tissue repair results from the combined contribution of donor xenogeneic chondrocytes and recruited host-derived cells present within the repair tissue, whose contribution to tissue repair—whether passive or stimulated by implanted cells—could not be determined from the present data [8,41,42]. This interpretation is supported by the work of Gelse et al., who implanted chondrocytes in the knee of miniature pigs and demonstrated that approximately 30% of cells within the repair tissue remained labeled, suggesting that most of the newly formed cartilage originated from the host-derived cells rather than from the implanted chondrocytes [46].

In the context of this cooperative repair process, it should be noted that the antibody for COL2A1 immunostaining used in our study is not species-specific and cross-reacts with rabbit collagen. As a result, we were unable to distinguish between the exogenously or endogenously produced collagen in the repaired tissue, which precludes a definitive attribution of ECM production to a specific cellular origin—whether donor-derived or host derived. This cross-reactivity is not a methodological oversight but reflects the high degree of evolutionary conservation of COL2A1 between species: pairwise sequence alignment of human (UniProt P02458) and rabbit (UniProt G1T5V9) COL2A1 reveals 97.1% amino acid identity (1444/1487 residues), a conservation that is explicitly acknowledged in the datasheets of all major commercially available anti-COL2A1 antibodies, none of which offer species discrimination at the protein level between human and rabbit. While these findings support the contribution of direct cell engraftment to cartilage repair, the potential role of indirect mechanisms remains an open question that future studies with appropriate experimental controls should address.

## 4. Materials and Methods

This study was conducted in accordance with the principles outlined in the Declaration of Helsinki. For the human procedures involving cartilage harvesting and chondrocyte culture, ethical approval was obtained from the Ethics Committee of EpiCURA hospital where the cartilage fragments were collected (P2022/031). Human articular cartilage fragments were obtained during knee replacement surgery performed for osteoarthritis. Written informed consent was obtained from all donors prior to sample collection. Clinical characteristics of the donors are presented in Table A1—Appendix E.

Animal experiments (rabbit surgeries) were approved by the institutional animal ethic committee (CEBEA, Comité Ethique du Bien-Etre Animal—Université libre de Bruxelles, protocol 702N).

**Figure 14:**
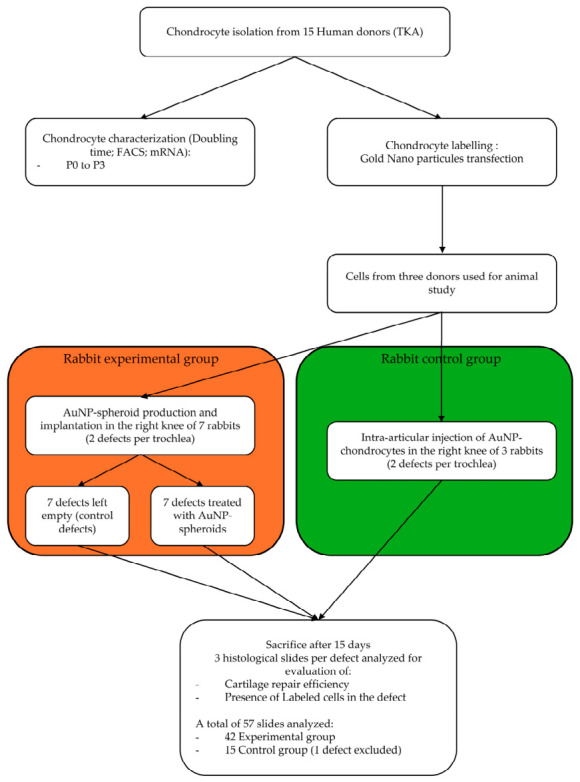
Flowchart of the study.

The flowchart (Figure 14) describes the study’s protocol. In summary, human chondrocytes were isolated from osteoarthritic knee cartilage and further expanded in vitro. Cells were labeled with 10 nm diameter silica-coated AuNPs using the polymer-based transfection agent SusFexin TS316 (Lamda Biotech, St. Louis, MO, USA). Chondrocytes were analyzed for viability, phenotype, proliferation, and extracellular matrix (ECM) gene as well as protein expression before and after AuNP transfection.

Two surgical trochlear defects were performed in the right knee of 10 rabbits.

Three rabbits then received an intra-articular injection of labeled chondrocytes in suspension (referred to as the rabbit control group).

In the rabbit experimental group (7 rabbits), spheroids of labeled chondrocytes were implanted in one of the two defects (referred to as treated defect) and the other one was left empty (referred to as control defect). After 2 weeks, the animals were sacrificed and cartilage repair, cell retention, and distribution were assessed (see Section 4.5 for details).

### 4.1. Isolation and Culture of Chondrocytes

Under the approval and guidance of the Ethics Committee and with informed consent from all patients, human articular cartilage was obtained from 15 patients (6 Women—9 Men) undergoing knee replacement surgery for osteoarthritis. The mean donor age was 63 years (range: 53–78 years). Cartilage samples were harvested from macroscopically unaffected regions of the knee joint, including the posterior femoral condyles, the lateral tibial plateau, and the trochlear groove.

Cartilage samples were washed and transported to the laboratory for processing in sterile phosphate-buffered saline (PBS) containing 200 U/mL penicillin and 200 μg/mL streptomycin (Invitrogen, Gent, Belgium).

Pieces of cartilage were rinsed to remove contaminants (synovial fluid, blood, adipose cells,…) and minced using a scalpel into approximately 1 mm^3^ fragments that were further subjected to overnight collagenase digestion in alpha-MEM/Ham’s F-12 (*v*/*v*) medium (Invitrogen, Gent, Belgium) containing 4 mM L-glutamine, 200 U/mL penicillin, 200 μg/mL streptomycin, and type II collagenase (0.15% *w*/*v*) (Cat. no: 0000175505, Sigma-Aldrich, Diegem, Belgium) at 37 °C in a humidified atmosphere with 5% CO_2_.

The resulting cell suspension was filtered through a 70 μm cell strainer to eliminate undigested tissue and rinsed with D-PBS to remove collagenase. Chondrocytes were counted and seeded at a density of 1 × 10^3^ cells/cm^2^ in culture flasks (25 to 75 cm^2^; cell monolayer passage 0, P0). Cells were cultured in the same medium [alpha-MEM/Ham’s F-12 (*v*/*v*) medium (Invitrogen, Gent, Belgium) containing 4 mM L-glutamine, 100 U/mL penicillin, 100 μg/mL streptomycin] supplemented with 10% human heat-inactivated serum (Serana Europe GmbH, Pessin, Germany; Cat. no: S-HU-EU-0125). No growth factors or supplements were added. Sterile filtration of the culture medium was performed using a Minisart NML Plus syringe filter (ref. 17823, Sartorius Stedim Biotech GmbH, Göttingen, Germany). The culture medium was changed twice weekly. Chondrocytes were enzymatically detached (Trypsin-EDTA; Invitrogen, Gent, Belgium) after 6–7 days of culture and reseeded at a density of 1 × 10^3^ cells/cm^2^ for a maximum of 3 monolayer passages (P1 to P3).

### 4.2. Chondrocyte Characterization

To characterize the isolated chondrocytes along cell passages and after transfection, several parameters were analyzed: cell population doubling time (DT), viability, and expression of chondrocyte genes, phenotypic markers, and proteins.

To evaluate the cell population doubling time (DT), 3 to 5 × 10^3^ chondrocytes were plated in 96-well plates and harvested when reaching 80–90% confluency. Cells were counted and further replated. DT was calculated using the formula DT = T × ln (2)⁄ln (ȼ2⁄ȼ1) where ȼ1 is the number of plated cells, ȼ2 is the number of harvested cells at the end of the culture period, and T is the time of culture (expressed in days).

To assess cell viability, chondrocytes were cultured in 96-well plates (3 to 5 × 10^3^ cells/well) for 1–5 days. Cell viability was evaluated after 15 min incubation with the nuclear-binding dyes propidium iodide (10 µg/mL; Sigma-Aldrich, Diegem, Belgium) and Hoechst 33342 (10 µg/mL; Sigma-Aldrich, Diegem, Belgium) using inverted microscopy (Axiovision Zeiss, Zaventem, Belgium) under UV light (excitation/emission wavelengths: 365/397 nm). Viable cells were identified by their blue-fluorescent intact nuclei, while non-viable cells showed yellow-red fluorescence. At least 500 cells were counted for each experimental condition.

Reverse transcription and quantitative polymerase chain reaction (RT-qPCR) were performed to evaluate the expression levels of aggrecan (ACAN), type II collagen (COL2A1), type I collagen (COL1A1), SRY-box transcription factor 9 (SOX-9), integrin α10β1 (ITG10), and integrin α11β1 (ITG11) mRNAs, which are indicative markers of chondrogenic potential. Total RNA was isolated from chondrocytes (P0 to P3) using the ReliaPrep™ system (Promega, Leiden, The Netherlands) following the manufacturer’s instructions. For each sample, 100 ng of RNA was reverse transcribed into cDNA in a total volume of 20 µL using the iScript™ cDNA Synthesis Kit (Bio-Rad, Nazareth Eke, Nazareth, Belgium). qPCR was then carried out using a CFX96 Real-Time PCR Detection System (Bio-Rad, Nazareth Eke, Nazareth, Belgium) in a total reaction volume of 20 µL, containing 3 mM MgCl_2_, 0.3 µM of each forward and reverse primer (Eurogentec, Seraing, Belgium—Table 2), 10 µL of SYBR Green Master Mix (Bio-Rad), and 2 µL of cDNA template. The thermal profile consisted of an initial denaturation at 95 °C for 3 min, followed by 40 cycles of 95 °C for 30 s and 60 °C for 30 s for denaturation and annealing/extension, respectively. Hypoxanthine phosphoribosyltransferase 1 (HPRT1) served as endogenous gene control.

Relative gene expression was quantified using the 2^−ΔΔCt^ method. The ΔCt was calculated by subtracting the cycle threshold (Ct) value of the reference gene (HPRT1) from that of the target gene. ΔΔCt values were then obtained by comparing the ΔCt of each passage (P1 to P3) to that of passage 0 (P0). Gene expression levels were expressed as fold changes relative to P0.

Expression of chondrocyte cell surface proteins was analyzed using fluorescence-activated cell sorting (FACS) immediately after cell isolation and after cell culture at each subsequent passage. The antibodies against human surface antigen were as follows: CD44-FITC (ab27285) from ABCAM, CD34-PE (ref 555822), CD45-FITC (ref 345808), CD49c-BV785 (ref 744521), CD54-PE (ref 555511), CD105-PerCP-Cy5-5 (ref 560819), CD166-BV421 (ref 562936) from BD Biosciences, and CD90-APC (FAB2067A) from R&D Systems. Each antibody was diluted according to the manufacturer’s recommendations. Data were acquired using BD FACS LSR Fortessa X-20 flow cytometers and processed using BD FACS Diva software v5.0.2.

Expression of ACAN and COL2A1 was assessed by immunohistochemistry (IHC) on cultured chondrocytes. Briefly, 3 to 5 × 10^3^ chondrocytes were seeded onto sterile glass coverslips and cultured under standard conditions until reaching the desired confluence. Cells were then fixed with paraformaldehyde, permeabilized, and blocked to prevent non-specific binding. Immunostaining was performed using primary antibodies against ACAN (Anti-Human Aggrecan Mouse Monoclonal Antibody [clone: HAG7D4], Origene, MD, USA) and COL2A1 (Anti-Human Collagen Type II Mouse Monoclonal Antibody [clone: II-4C11], MP Bio-medicals, CA, USA) diluted 1/250 in TBS containing 1% normal horse serum, followed by appropriate secondary antibodies (Anti-Mouse IgG biotinylated—diluted 1/100 in TBS + normal horse serum 1%). Signal detection was achieved using the avidin–biotin complex (ABC) method, according to the manufacturer’s instructions, as previously described for COL2A1staining (Appendix F: IHC protocol).

### 4.3. Gold Nanoparticles Labelling of Chondrocytes and Impact on Cell Functions

Chondrocytes at P1 were seeded at a density of 14 × 10^3^ cells/cm^2^ for AuNPs labeling. Upon reaching approximately 80% confluence, each T25 cm^2^ culture flask was rinsed twice with serum-free DMEM (Invitrogen, Ghent, Belgium) to remove residual serum. The transfection mixture was prepared by adding 32 µL of SusFexin (Lamda Biotech, St. Louis, USA) to 590 µL of serum-free DMEM. After gentle mixing, 96 µL of silica-coated AuNPs (10 nm diameter; Sigma-Aldrich, Saint-Quentin-Fallavier Cedex, France) were added to the solution. The mixture was incubated at room temperature for 15 min to allow complex formation between SusFexin and AuNPs. Subsequently, 5.6 mL of DMEM was added to the transfection mixture, and the entire volume was carefully applied to each T25 cm^2^ culture; cells were then incubated at 37 °C in a 5% CO_2_ atmosphere for 24 h. For the control, non-transfected cells, 5.6 mL of DMEM serum free medium was added for 24 h. After transfection, chondrocytes were characterized to ensure cellular biology preservation (see Section 4.2).

To determine the efficiency of transfection, visualization of the chondrocytes labeled with AuNPs was performed using a Silver Enhancer Kit (SE100; Sigma-Aldrich Diegem, Belgium) in accordance with the manufacturer’s protocol, enabling enhanced contrast under light microscopy. Hematoxylin and safranin-O staining were then performed on samples for contrast enhancement in light microscopy. This method enabled the distinction between AuNP-positive and -negative labeled chondrocytes and provided a semi-quantitative estimate of the AuNPs’ internalization efficiency based on the presence of silver-enhanced signal.

### 4.4. Spheroids Production

To generate spheroids, 96-well plates (flat-bottom) were coated with low EEO agarose 2% *w*/*v* (Cat. No. J66369.18; Thermo Fisher, Aalst, Belgium) melted in cell culture medium (2% *w*/*v*). AuNP-labeled chondrocytes at P1 were collected, counted, and seeded at a density of 15 × 10^3^–20 × 10^3^ cells per well and cultured under the conditions described above. During that culture period, the cells aggregated to form a three-dimensional (3D) spheroid structure. After two weeks, three individual spheroids were collected and grouped into a coated single well to promote their fusion. A large spheroid containing approximately 60 × 10^3^ cells was obtained after a further culture period of 7 days.

### 4.5. Rabbit Surgeries

Ten female 15-week-old New Zealand white rabbits weighing 3.0 to 3.5 kg (CER Group, Marloie, Belgium—LA1800104) were randomly divided into two groups: one group of seven rabbits for spheroid implantation (experimental group), and one group of three rabbits receiving an intra articular injection of AuNP-labeled free chondrocytes (control group). The right knee of both rabbit groups was operated on to create two articular lesions of the trochlea (two holes of 4 mm Ø; see below). All surgeries were conducted under aseptic conditions in a dedicated animal surgery room. Rabbits were anesthetized via subcutaneous injection of ketamine (30 mg/kg) and xylazine (5 mg/kg). For analgesia, they received an intramuscular injection of buprenorphine (0.05 mg/kg) during surgery.

A longitudinal cutaneous incision centered over the patella was made, followed by an internal arthrotomy with external luxation of the patella to expose the trochlea. Two defects, each 4 mm in diameter, were created by removing the full thickness of cartilage while preserving the subchondral bone.

**Figure 15:**
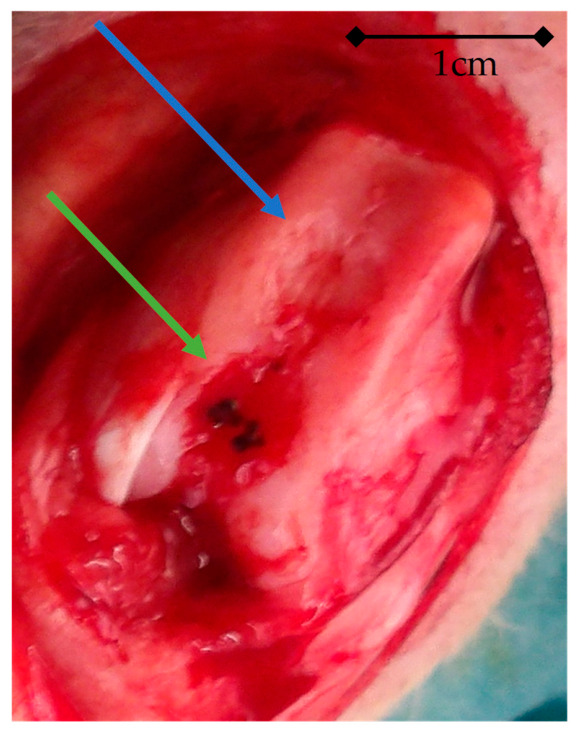
Intraoperative image of the trochlea treated with 6 spheroids applied to the inferior defect (green arrow). The superior defect was left empty (blue arrow).

In the rabbit experimental group, one lesion was filled with spheroids, and the second lesion was left empty and used as a control defect (Figure 15). The number of spheroids used for implantation was similar to the current recommendations in clinical practice: 4 to 6 spheroids (each containing approximatively 45 × 10^3^–60 × 10^3^ cells) were implanted in each defect of Ø 4 mm [38].

In the rabbit control group receiving an intra-articular injection of free chondrocytes, the patella was repositioned, and the joint capsule and medial femoro-patellar ligament were sutured. Subsequently, 1 × 10^6^ AuNP-chondrocytes were injected through the articular capsule using a 23-gauge needle.

It should be noted that the design of the two groups differed inherently in their control structure. In the experimental group, one defect—left empty and untreated—served as a dedicated internal control, allowing for direct comparison between treated and untreated repair within the same animal and the same joint. In the intra-articular injection group, however, no equivalent internal control could be designated. Since AuNP-labeled chondrocytes were injected through the closed joint capsule without targeting either defect specifically, it was not possible to determine whether injected cells migrated preferentially to one or both lesions. This delivery route was intentionally chosen to replicate current clinical practice (intra-articular injections). Accordingly, both defects in the injection group were analyzed as treated defects.

After surgery, all rabbits were allowed to move freely without the use of splints and were housed individually. Animals were sacrificed two weeks after surgery to evaluate cartilage repair and compare the efficacy of each cell delivery method.

### 4.6. Evaluation of the Persistence of Implanted Cells in the Trochlear Defect

To assess the engraftment and persistence of transplanted cells in the knee, the presence of AuNPs-labeled chondrocytes was evaluated by silver enhancement staining on tissue sections obtained from both the experimental and control rabbit groups.

Following sacrifice, rabbit knees were recovered, fixed in 4% paraformaldehyde for 3 days, and then stored in phosphate-buffered saline (PBS) at 4 °C. Subsequently, samples were decalcified in 12.5% ethylenediaminetetraacetic acid (EDTA; Avantor, The Netherlands) at pH 7.2 for 28 days. After thorough washing, tissues were embedded in paraffin. Serial sections of 4 μm thickness were cut and mounted on Superfrost Plus™ slides (Thermo Scientific, Waltham, MA, USA) for each hole performed during surgery (Center for Microscopy and Molecular Imaging (CMMI), Gosselies, Belgium).

Visualization of chondrocytes labeled with AuNPs was performed using a Silver Enhancer Kit (SE100; Sigma-Aldrich, Diegem, Belgium) in accordance with the manufacturer’s protocol, enabling enhanced contrast under light microscopy. Briefly, the histological slides were rehydrated, rinsed in distilled water, and incubated with the silver enhancer mixture for 7 min at 4 °C. Subsequently, the slides were rinsed with distilled water and fixed with a 2.5% sodium thiosulfate solution. Hematoxylin and safranin-O staining was then performed, followed by dehydration and mounting in DPX.

### 4.7. Evaluation of Cartilage Repair

Macroscopic evaluation of cartilage defect healing was performed at the time of sacrifice using the ICRS macroscopic scoring system [51]. This standardized assessment allowed for semi-quantitative comparison of repair quality across animal groups and defect groups based on surface integrity, color, and integration with surrounding cartilage. As this system was originally designed for human joints, the criterion for an “integration border zone” was adapted to the anatomical scale of the rabbit knee [18].

The IHC protocol for COL2A1 was performed as described above for each trochlear defect to evaluate histological cartilage repair.

Histological cartilage repair was categorized into three grades: (1) no repair (absence of tissue filling within the defect); (2) fibrocartilaginous repair (filling with tissue lacking COL2A1); and (3) partial hyaline differentiation (presence of COL2A1 in the repair tissue). In addition, detailed histological assessment was performed using the modified O’Driscoll scoring system [52,53]. Mean total scores were compared between trochlear defects of each group (AuNP free chondrocytes; AuNP spheroids and control defects). For item 1 (cell morphology), when there was a heterogeneous tissue, the most differentiated tissue was considered for scoring. The maximum total score was 23 points.

### 4.8. Statistics

Statistical analysis was performed using R software (R Core Team, Vienna, Austria, 2024). Continuous variables are reported as means ± standard error (SEM). Categorical variables are expressed as counts with percentages and odds ratios.

Normality of all continuous variables was assessed using the Shapiro–Wilk test. Since no variable met the normality assumption, non-parametric tests were used throughout the analysis. Comparisons of independent quantitative variables between more than two groups (ICRS and O’Driscoll scores) were conducted using the Kruskal–Wallis test (used for independent groups in the absence of normal distribution). In cases of repeated measures or matched samples across groups (doubling time; mRNA/qPCR over passages), the Friedman test was employed. When the global test was significant, post hoc pairwise comparisons were performed using Dunn’s test with Bonferroni correction for multiple testing.

For categorical variables, the generalized Fisher’s exact test (Fisher–Freeman–Halton test) was used for 3 × 3 contingency tables [54]. Pairwise group comparisons were then assessed by repeating Fisher’s exact tests to identify specific differences. When significant, pairwise group comparisons were further assessed by individual Fisher’s exact tests to identify specific differences.

Macroscopic (ICRS) and microscopic (O’Driscoll score) evaluations were independently graded by two blinded orthopedic surgeons.

Statistical significance was set at *p* < 0.05 for all analyses.

## 5. Conclusions

This study demonstrates that human chondrocytes implanted in the form of spheroids can successfully engraft into articular cartilage defects and initiate early regenerative processes in a rabbit xenogeneic model without the need for immunosuppression. Compared with intra-articular injection of free chondrocytes, spheroid implantation was associated with improved cell retention at the defect site and better structural filling. Localized expression of cartilage-specific ECM components was also observed, although only in 28% of spheroid-treated defects (2 out of 7), and the species origin of the COL2A1 signal—human donor or rabbit host—could not be determined. These findings are consistent with early chondrogenic activity but are insufficient to establish hyaline cartilage regeneration at this short-term timepoint.

The use of AuNPs allowed for effective cell tracking and might offer additional anti-inflammatory benefits in future therapeutic applications. The partial repair observed in untreated defects suggests that spheroids may also exert paracrine effects that enhance host-driven regeneration.

Altogether, our findings support the clinical potential of spheroid-based approaches for focal cartilage repair, and that the rabbit xenogeneic model represents a useful pre-clinical platform for evaluating human-derived cell therapies. Future studies should explore long-term functional outcomes, immune responses, and the role of extracellular vesicles and other paracrine mediators in enhancing cartilage regeneration.

## Figures and Tables

**Table 1:** Results of cartilage repair: Microscopic evaluation (histological cartilage repair groups) and O’Driscoll score.

	Control Group *n* = 5	Experimental Group		*p*
		Spheroids *n* = 7	Left Empty *n* = 7	
Histological cartilage repair groups				
No repair	4 (80%)	2 (28.6%)	2 (28.6%)	0.21
Pure fibrocartilage	1 (20%)	3 (42.8%)	5 (71.4%)	
Partial hyaline tissue	0	2 (28.6%)	0 (0%)	
O’Driscoll	5.9 ± 0.7	12.6 ± 0.96	12.4 ± 0.9	0.00011 *

* O’Driscoll post hoc pairwise Dunn test with Bonferroni correction: Spheroid-treated vs. Control group, *p* = 0.0004; Left empty vs. Control group, *p* = 0.0006; Spheroid-treated vs. Left empty, *p* = 1.00.

**Table 2:** Primer sequences for real-time qPCR.

Gene	Amplicon Size (bp)	Oligonucleotide (5′ -> 3′)	
		Forward	Reverse
hCol2A1	160	5′ GCTGGTGCCTCCGGTAAC 3′	5′ CACCCGTCTGACCTTTCGG 3′
hACAN	185	5′ CCAGGAGGTATGTGAGGA 3′	5′ CGATCCACTGGTAGTCTTG 3′
hCol1A1	99	5′ CGAAGACATCCCACCAATCAC 3′	5′ TTGTCGCAGACGCAGATCC 3′
hITG10	101	5′ TGGACTATTGAGGCTGGTTCAC 3′	5′ CACTGCCCTCTTCGGTTCC 3′
hITG11	176	5′ GCACGACATCAGTGGCAATAAG 3′	5′ CGAGGCGCATGTTGTCTTTC 3′
hSOX-9	85	5′ AGCGAACGCACATCAAGAC 3′	5′ CTGTAGGCGATCTGTTGGGG 3′
hHPRT1	189	5′ GGCGTCGTGATTAGTGATGAT 3′	5′ CTTGAGCACACAGAGGGCTAC 3′

**Figure A1:**
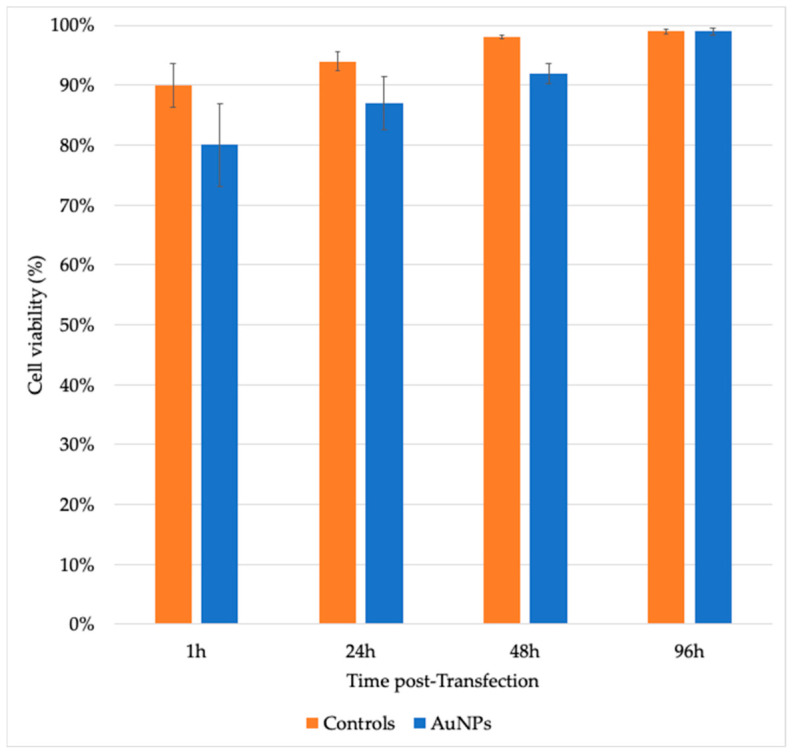
Viability of AuNP-labeled and -unlabeled control chondrocytes. No statistical difference (*n* = 5; *p* > 0.05).

**Figure A2:**
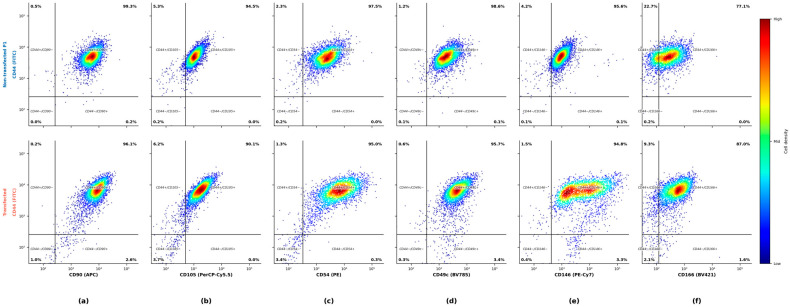
Representative flow cytometry plots of P1 chondrocytes labeled and unlabeled, cultured in 10% human serum. The plots depict the co-expression of CD44 with specific surface markers, identifying subpopulations as follows: CD44^+^/CD90^+^ (**a**), CD44^+^/CD105^+^ (**b**), CD44^+^/CD54^+^ (**c**), CD44^+^/CD49c^+^ (**d**), CD44^+^/CD146^+^ (**e**), and CD44^+^/CD166^+^ (**f**).

**Figure A3:**
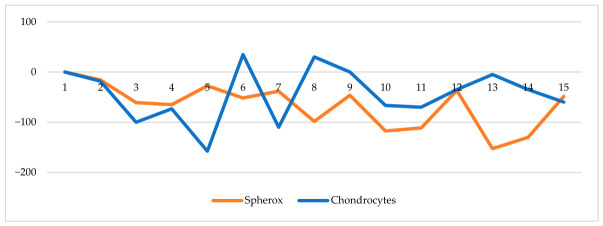
Evolution of weight loss compared to J0 over study time.

**Figure A4:**
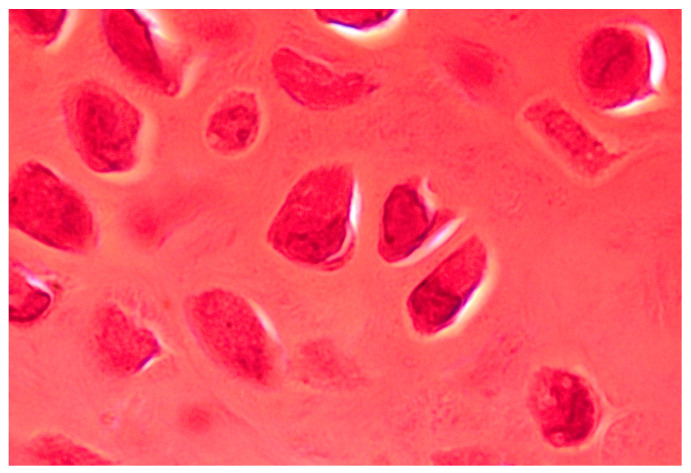
Absence of AuNP-positive cells in the repair tissue of a defect in the control group (Safranin-O staining). Zoom = 80×—immersion.

## Data Availability

The data presented in this study are available on request from the corresponding author. The data are not publicly available due to privacy restrictions related to patient data.

## Supplementary Materials

The following supporting information can be downloaded at: https://www.mdpi.com/article/10.3390/ijms27146381/s1.

## Abbreviations

The following abbreviations are used in this manuscript:

α-MEM	Alpha Minimum Essential Medium
ACAN	Aggrecan
APC	Allophycocyanin
AuNPs	Gold Nanoparticles
BMP-2	Bone Morphogenetic Protein-2
BV421	Brilliant Violet 421
BV785	Brilliant Violet 785
CO_2_	Carbon Dioxide
COL1A1	Collagen Type I Alpha 1
COL2A1	Collagen Type II Alpha 1
cDNA	Complementary DNA
DMEM	Dulbecco’s Modified Eagle Medium
DNA	Deoxyribonucleic Acid
D-PBS	Dulbecco’s Phosphate Buffered Salin
DT	Doubling Time
EDTA	Ethylenediaminetetraacetic Acid
ECM	Extracellular Matrix
EVs	Extra Vesicles
FACS	Fluorescence-Activated Cell Sorting
FBS	Fetal Bovine Serum
FITC	Fluorescein Isothiocyanate
HS	Human Serum
HPRT1	Hypoxanthine-Guanine Phosphoribosyltransferase 1
ICRS	International Cartilage Repair Society
IHC	Immunohistochemistry
min	Minute
MMP-13	Matrix Metalloproteinase-13
mRNA	Messenger Ribonucleic Acid
MSC	Mesenchymal Stem Cell
NC3Rs	National Centre for the Replacement, Refinement and Reduction of Animals in Research
OA	Osteoarthritis
OD	O’Driscoll
PBS	Phosphate Buffered Saline
PE	Phycoerythrin
PerCP-Cy5	Peridinin-Chlorophyll-Protein-Cyanine5
Px	Passage (x = number of the passage)
PCR	Polymerase Chain Reaction
qPCR/RT-qPCR	Quantitative Polymerase Chain Reaction/Real-Time Quantitative PCR
TBS	Tris-Buffered Saline
TKA	Total Knee Arthroplasty
UV	Ultraviolet

## Appendix E

**Table A1:** Donors’ clinical characteristics (in gray are the three donors used for rabbit surgery).

Sex	Age	BMI	Smoking	Alcohol	Hard Drug	Hypertension	Diabetes	Other
F_1_	78	29	No	No	No	Yes	No	
M_1_	62	35	No	Yes	No	Yes	No	
F_2_	56	32	Yes	No	No	Yes	No	
F_3_	76	33	No	Yes	No	Yes	No	
M_2_	74	30	No	Yes	No	Yes	Yes	Renal failure
M_3_	58	38	Yes	Yes	Yes	No	No	
F_4_	60	32	Yes	No	No	Yes	Yes	
M_4_	69	44	Yes	No	No	No	No	
F_5_	60	33	No	No	No	Yes	Yes	
M_5_	58	30	No	No	No	Yes	No	Post-traumatic arthrosis
F_6_	59	28	No	No	No	No	No	
M_6_	62	33	No	No	No	Yes	No	
M_7_	62	24	Yes	Yes	No	No	No	Cirrhosis
M_8_	63	25	No	No	No	No	No	
M_9_	53	38	Yes	No	No	Yes	No	Hypercholesterolemia

## Appendix F. Immunohistochemistry (IHC) Protocol for Rabbit

Briefly, slides were warmed at 60 °C for 30 min and then dewaxed by toluene (two baths) and 100% alcohol (two baths). After a 30 min treatment with H_2_O_2_ (0.3%) in methanol to inhibit endogenous peroxidase, the slides were washed for 5 min in Tris buffer saline (TBS; 0.01 mol/L Tris, 0.15 mol/L NaCl, pH 7.4) and incubated at 37 °C in trypsin (0.1% in TBS) for 30 min. After three washings (10 min each) in TBS, the slides were incubated with the blocking solution (10%, *v*/*v* normal horse serum in TBS) [18].

After overnight incubation at room temperature with the primary antibody (ACAN or COL2A1) diluted 1/250 in TBS containing 1% normal horse serum, the slides were washed in TBS (3 × 5 min) and sequentially incubated with secondary antibody conjugated to biotin (diluted 1/100 in TBS + normal horse serum 1%). Slides were washed again (TBS 3 × 5 min), and the signal amplified by the ABC Kit (Vector, San Mateo, CA, USA) for 30 min and washed (TBS 3 × 5 min). The peroxidase activity was revealed using diaminobenzidine (DACO) as the chromogen and stopped with distilled water [55]. The slides were counterstained with hematoxylin [56] before dehydration in croissant baths of alcohol followed by two baths of toluene; they were then mounted in D.P.X. (Sigma-Aldrich, St. Louis, MO, USA).

## References

[1] Sophia Fox A.J., Bedi A., Rodeo S.A. (2009). The Basic Science of Articular Cartilage: Structure, Composition, and Function. Sports Health A Multidiscip. Approach.

[2] Ross K.A., Ferati S.R., Alaia M.J., Kennedy J.G., Strauss E.J. (2024). Current and Emerging Techniques in Articular Cartilage Repair. Bull. Hosp. Jt. Dis..

[3] Jiang Y., Lin H., Tuan R.S. (2017). Overview: State of the Art and Future Prospectives for Cartilage Repair. Cartilage.

[4] Abraamyan T., Johnson A.J., Wiedrick J., Crawford D.C. (2022). Marrow Stimulation Has Relatively Inferior Patient-Reported Outcomes in Cartilage Restoration Surgery of the Knee: A Systematic Review and Meta-Analysis of Randomized Controlled Trials. Am. J. Sports Med..

[5] Krych A.J., Nawabi D.H., Farshad-Amacker N.A., Jones K.J., Maak T.G., Potter H.G., Williams R.J. (2016). Bone Marrow Concentrate Improves Early Cartilage Phase Maturation of a Scaffold Plug in the Knee: A Comparative Magnetic Resonance Imaging Analysis to Platelet-Rich Plasma and Control. Am. J. Sports Med..

[6] Bedi A., Feeley B.T., Williams R.J.I. (2010). Management of Articular Cartilage Defects of the Knee. J. Bone Jt. Surg..

[7] Vinod E., Boopalan P.R.J.V.C., Sathishkumar S. (2018). Reserve or Resident Progenitors in Cartilage? Comparative Analysis of Chondrocytes versus Chondroprogenitors and Their Role in Cartilage Repair. Cartilage.

[8] De Moor L., Beyls E., Declercq H. (2020). Scaffold Free Microtissue Formation for Enhanced Cartilage Repair. Ann. Biomed. Eng..

[9] Vonk L.A., Roël G., Hernigou J., Kaps C., Hernigou P. (2021). Role of Matrix-Associated Autologous Chondrocyte Implantation with Spheroids in the Treatment of Large Chondral Defects in the Knee: A Systematic Review. Int. J. Mol. Sci..

[10] Anderer U., Libera J. (2002). In Vitro Engineering of Human Autogenous Cartilage. J. Bone Miner. Res..

[11] Schubert T., Anders S., Neumann E., Schölmerich J., Hofstädter F., Grifka J., Müller-Ladner U., Libera J., Schedel J. (2009). Long-Term Effects of Chondrospheres on Cartilage Lesions in an Autologous Chondrocyte Implantation Model as Investigated in the SCID Mouse Model. Int. J. Mol. Med..

[12] Smith B., Sigal I.R., Grande D.A. (2015). Immunology and Cartilage Regeneration. Immunol. Res..

[13] Mage R.G., Esteves P.J., Rader C. (2019). Rabbit Models of Human Diseases for Diagnostics and Therapeutics Development. Dev. Comp. Immunol..

[14] Soares J., Pinheiro A., Esteves P.J. (2022). The Rabbit as an Animal Model to Study Innate Immunity Genes: Is It Better than Mice?. Front. Immunol..

[15] Ramallal M., Maneiro E., López E., Fuentes-Boquete I., López-Armada M.J., Fernández-Sueiro J.L., Galdo F., De Toro F.J., Blanco F.J. (2004). Xeno-Implantation of Pig Chondrocytes into Rabbit to Treat Localized Articular Cartilage Defects: An Animal Model. Wound Repair Regen..

[16] Zhao F., Zhao Y., Liu Y., Chang X., Chen C., Zhao Y. (2011). Cellular Uptake, Intracellular Trafficking, and Cytotoxicity of Nanomaterials. Small.

[17] Grässel S., Aszódi A. (2017). Cartilage.

[18] Hernigou J., Vertongen P., Chahidi E., Kyriakidis T., Dehoux J.-P., Crutzen M., Boutry S., Larbanoix L., Houben S., Gaspard N. (2018). Effects of Press-Fit Biphasic (Collagen and HA/βTCP) Scaffold with Cell-Based Therapy on Cartilage and Subchondral Bone Repair Knee Defect in Rabbits. Int. Orthop..

[19] Wakitani S., Goto T., Pineda S.J., Young R.G., Mansour J.M., Caplan A.I., Goldberg V.M. (1994). Mesenchymal Cell-Based Repair of Large, Full-Thickness Defects of Articular Cartilage. J. Bone Jt. Surg. Am..

[20] Ikebe S., Masumi S., Yano H., Fukunaga T., Shimizu K., Shin S. (1996). Immunosuppressive Effect of Tacrolimus (FK-506). Bone Xenografts in Rabbits. Acta Orthop. Scand..

[21] Jang K.-M., Lee J.-H., Park C.M., Song H.-R., Wang J.H. (2014). Xenotransplantation of Human Mesenchymal Stem Cells for Repair of Osteochondral Defects in Rabbits Using Osteochondral Biphasic Composite Constructs. Knee Surg. Sports Traumatol. Arthrosc..

[22] Pei M., Yan Z., Shoukry M., Boyce B.M. (2010). Failure of Xenoimplantation Using Porcine Synovium-Derived Stem Cell-Based Cartilage Tissue Constructs for the Repair of Rabbit Osteochondral Defects. J. Orthop. Res..

[23] Caron M.M.J., Emans P.J., Coolsen M.M.E., Voss L., Surtel D.a.M., Cremers A., van Rhijn L.W., Welting T.J.M. (2012). Redifferentiation of Dedifferentiated Human Articular Chondrocytes: Comparison of 2D and 3D Cultures. Osteoarthr. Cartil..

[24] Balfourier A., Luciani N., Wang G., Lelong G., Ersen O., Khelfa A., Alloyeau D., Gazeau F., Carn F. (2020). Unexpected Intracellular Biodegradation and Recrystallization of Gold Nanoparticles. Proc. Natl. Acad. Sci. USA.

[25] Wallenborn M., Petters O., Rudolf D., Hantmann H., Richter M., Ahnert P., Rohani L., Smink J.J., Bulwin G.C., Krupp W. (2018). Comprehensive High-Resolution Genomic Profiling and Cytogenetics of Human Chondrocyte Cultures by GTG-Banding, Locus-Specific FISH, SKY and SNP Array. Eur. Cells Mater..

[26] Yin W., Qian Z., Liu C., Song B., Sun Q. (2025). Autologous Serum Supplementation Promotes the Phenotype Maintenance of Human Chondrocytes with Increased Cellular Autophagy. Sci. Rep..

[27] Lin Z., Fitzgerald J.B., Xu J., Willers C., Wood D., Grodzinsky A.J., Zheng M.H. (2008). Gene Expression Profiles of Human Chondrocytes During Passaged Monolayer Cultivation. J. Orthop. Res..

[28] Varas L., Ohlsson L.B., Honeth G., Olsson A., Bengtsson T., Wiberg C., Bockermann R., Järnum S., Richter J., Pennington D. (2007). Alpha10 Integrin Expression Is Up-Regulated on Fibroblast Growth Factor-2-Treated Mesenchymal Stem Cells with Improved Chondrogenic Differentiation Potential. Stem Cells Dev..

[29] Bengtsson T., Aszodi A., Nicolae C., Hunziker E.B., Lundgren-Akerlund E., Fässler R. (2005). Loss of Alpha10beta1 Integrin Expression Leads to Moderate Dysfunction of Growth Plate Chondrocytes. J. Cell Sci..

[30] Lundgren-Åkerlund E., Aszòdi A. (2014). Integrin A10β1: A Collagen Receptor Critical in Skeletal Development. Adv. Exp. Med. Biol..

[31] Wenke A.-K., Rothhammer T., Moser M., Bosserhoff A.K. (2006). Regulation of Integrin Alpha10 Expression in Chondrocytes by the Transcription Factors AP-2epsilon and Ets-1. Biochem. Biophys. Res. Commun..

[32] Rikkers M., Korpershoek J.V., Levato R., Malda J., Vonk L.A. (2021). Progenitor Cells in Healthy and Osteoarthritic Human Cartilage Have Extensive Culture Expansion Capacity While Retaining Chondrogenic Properties. Cartilage.

[33] Rikkers M., Korpershoek J.V., Levato R., Malda J., Vonk L.A. (2022). The Clinical Potential of Articular Cartilage-Derived Progenitor Cells: A Systematic Review. npj Regen. Med..

[34] Pettenuzzo S., Arduino A., Belluzzi E., Pozzuoli A., Fontanella C.G., Ruggieri P., Salomoni V., Majorana C., Berardo A. (2023). Biomechanics of Chondrocytes and Chondrons in Healthy Conditions and Osteoarthritis: A Review of the Mechanical Characterisations at the Microscale. Biomedicines.

[35] Pan Y., Neuss S., Leifert A., Fischler M., Wen F., Simon U., Schmid G., Brandau W., Jahnen-Dechent W. (2007). Size-Dependent Cytotoxicity of Gold Nanoparticles. Small.

[36] Pascarelli N.A., Moretti E., Terzuoli G., Lamboglia A., Renieri T., Fioravanti A., Collodel G. (2013). Effects of Gold and Silver Nanoparticles in Cultured Human Osteoarthritic Chondrocytes. J. Appl. Toxicol..

[37] Hoburg A., Niemeyer P., Laute V., Zinser W., Becher C., Kolombe T., Fay J., Pietsch S., Kuźma T., Widuchowski W. (2023). Sustained Superiority in KOOS Subscores after Matrix-Associated Chondrocyte Implantation Using Spheroids Compared to Microfracture. Knee Surg. Sports Traumatol. Arthrosc..

[38] Hoburg A., Niemeyer P., Laute V., Zinser W., John T., Becher C., Izadpanah K., Diehl P., Kolombe T., Fay J. (2022). Safety and Efficacy of Matrix-Associated Autologous Chondrocyte Implantation with Spheroids for Patellofemoral or Tibiofemoral Defects: A 5-Year Follow-up of a Phase 2, Dose-Confirmation Trial. Orthop. J. Sports Med..

[39] Sumida Y., Nakamura K., Feil S., Siebold M., Kirsch J., Siebold R. (2022). Good Healing Potential of Patellar Chondral Defects After All-Arthroscopic Autologous Chondrocyte Implantation with Spheroids: A Second-Look Arthroscopic Assessment. Knee Surg. Sports Traumatol. Arthrosc..

[40] Jeon J.H., Yun B.G., Lim M.J., Kim S.J., Lim M.H., Lim J.Y., Park S.H., Kim S.W. (2020). Rapid Cartilage Regeneration of Spheroids Composed of Human Nasal Septum-Derived Chondrocyte in Rat Osteochondral Defect Model. Tissue Eng. Regen. Med..

[41] Omelyanenko N.P., Karalkin P.A., Bulanova E.A., Koudan E.V., Parfenov V.A., Rodionov S.A., Knyazeva A.D., Kasyanov V.A., Babichenko I.I., Chkadua T.Z. (2020). Extracellular Matrix Determines Biomechanical Properties of Chondrospheres During Their Maturation In Vitro. Cartilage.

[42] Imabayashi H., Mori T., Gojo S., Kiyono T., Sugiyama T., Irie R., Isogai T., Hata J., Toyama Y., Umezawa A. (2003). Redifferentiation of Dedifferentiated Chondrocytes and Chondrogenesis of Human Bone Marrow Stromal Cells via Chondrosphere Formation with Expression Profiling by Large-Scale cDNA Analysis. Exp. Cell Res..

[43] Mahmoudifar N., Doran P.M. (2006). Effect of Seeding and Bioreactor Culture Conditions on the Development of Human Tissue-Engineered Cartilage. Tissue Eng..

[44] Bartz C., Meixner M., Giesemann P., Roël G., Bulwin G.-C., Smink J.J. (2016). An Ex Vivo Human Cartilage Repair Model to Evaluate the Potency of a Cartilage Cell Transplant. J. Transl. Med..

[45] Benya P.D., Shaffer J.D. (1982). Dedifferentiated Chondrocytes Reexpress the Differentiated Collagen Phenotype When Cultured in Agarose Gels. Cell.

[46] Gelse K., Brem M., Klinger P., Hess A., Swoboda B., Hennig F., Olk A. (2009). Paracrine Effect of Transplanted Rib Chondrocyte Spheroids Supports Formation of Secondary Cartilage Repair Tissue. J. Orthop. Res..

[47] Vonk L.A. (2023). Potency Assay Considerations for Cartilage Repair, Osteoarthritis and Use of Extracellular Vesicles. Adv. Exp. Med. Biol..

[48] Mustonen A.-M., Nieminen P. (2021). Extracellular Vesicles and Their Potential Significance in the Pathogenesis and Treatment of Osteoarthritis. Pharmaceuticals.

[49] Wu K.-C., Yang H.-I., Chang Y.-H., Chiang R.Y.-S., Ding D.-C. (2025). Extracellular Vesicles Derived from Human Umbilical Mesenchymal Stem Cells Transfected with miR-7704 Improved Damaged Cartilage and Reduced Matrix Metallopeptidase 13. Cells.

[50] Scalzone A., Ferreira A.M., Tonda-Turo C., Ciardelli G., Dalgarno K., Gentile P. (2019). The Interplay Between Chondrocyte Spheroids and Mesenchymal Stem Cells Boosts Cartilage Regeneration Within a 3D Natural-Based Hydrogel. Sci. Rep..

[51] Mithoefer K., Saris D.B.F., Farr J., Kon E., Zaslav K., Cole B.J., Ranstam J., Yao J., Shive M., Levine D. (2011). Guidelines for the Design and Conduct of Clinical Studies in Knee Articular Cartilage Repair: International Cartilage Repair Society Recommendations Based on Current Scientific Evidence and Standards of Clinical Care. Cartilage.

[52] O’Driscoll S.W., Keeley F.W., Salter R.B. (1986). The Chondrogenic Potential of Free Autogenous Periosteal Grafts for Biological Resurfacing of Major Full-Thickness Defects in Joint Surfaces Under the Influence of Continuous Passive Motion. An Experimental Investigation in the Rabbit. J. Bone Jt. Surg. Am..

[53] Qi Y., Zhao T., Xu K., Dai T., Yan W. (2012). The Restoration of Full-Thickness Cartilage Defects with Mesenchymal Stem Cells (MSCs) Loaded and Cross-Linked Bilayer Collagen Scaffolds on Rabbit Model. Mol. Biol. Rep..

[54] Grayling M., Mander A., Wason J. (2017). A Two-Stage Fisher Exact Test for Multi-Arm Studies with Binary Outcome Variables. arXiv.

[55] Bondulich M.K., Guo T., Meehan C., Manion J., Rodriguez Martin T., Mitchell J.C., Hortobagyi T., Yankova N., Stygelbout V., Brion J.-P. (2016). Tauopathy Induced by Low Level Expression of a Human Brain-Derived Tau Fragment in Mice Is Rescued by Phenylbutyrate. Brain.

[56] Schmitz N., Laverty S., Kraus V.B., Aigner T. (2010). Basic Methods in Histopathology of Joint Tissues. Osteoarthr. Cartil..

